# Genetically enhanced T lymphocytes and the intensive care unit

**DOI:** 10.18632/oncotarget.24637

**Published:** 2018-03-27

**Authors:** Tiberiu Tat, Huming Li, Catalin-Sorin Constantinescu, Anca Onaciu, Sergiu Chira, Ciprian Osan, Sergiu Pasca, Bobe Petrushev, Vlad Moisoiu, Wilhelm-Thomas Micu, Cristian Berce, Sebastian Tranca, Delia Dima, Ioana Berindan-Neagoe, Jianliang Shen, Ciprian Tomuleasa, Liren Qian

**Affiliations:** ^1^ Intensive Care Unit, Ion Chiricuta Clinical Cancer Research, Cluj Napoca, Romania; ^2^ Department of Anesthesiology-Intensive Care, Iuliu Hatieganu University of Medicine and Pharmacy, Cluj Napoca, Romania; ^3^ Department of Pulmonary and Critical Care Medicine, Navy General Hospital of PLA, Beijing, China; ^4^ Department of Hematology, Iuliu Hatieganu University of Medicine and Pharmacy, Cluj Napoca, Romania; ^5^ Research Center for Functional Genomics and Translational Medicine, Iuliu Hatieganu University of Medicine and Pharmacy, Cluj Napoca, Romania; ^6^ Department of Experimental Medicine, Iuliu Hatieganu University of Medicine and Pharmacy, Cluj Napoca, Romania; ^7^ Department of Hematology, Ion Chiricuta Clinical Cancer Research, Cluj Napoca, Romania; ^8^ Department of Hematology, Navy General Hospital of PLA, Beijing, China; ^9^ Research Center for Functional Genomics and Translational Medicine / Hematology, Iuliu Hatieganu University of Medicine and Pharmacy, Cluj Napoca, Romania

**Keywords:** hematological malignancies, chimeric antigen receptor-modified T cell, donor lymphocyte infusion, stem cell transplant, immunotherapy

## Abstract

Chimeric antigen receptor-modified T cells (CAR-T cells) and donor lymphocyte infusion (DLI) are important protocols in lymphocyte engineering. CAR-T cells have emerged as a new modality for cancer immunotherapy due to their potential efficacy against hematological malignancies. These genetically modified receptors contain an antigen-binding moiety, a hinge region, a transmembrane domain, and an intracellular costimulatory domain resulting in lymphocyte T cell activation subsequent to antigen binding. In present-day medicine, four generations of CAR-T cells are described depending on the intracellular signaling domain number of T cell receptors. DLI represents a form of adoptive therapy used after hematopoietic stem cell transplant for its anti-tumor and anti-infectious properties. This article covers the current status of CAR-T cells and DLI research in the intensive care unit (ICU) patient, including the efficacy, toxicity, side effects and treatment.

## INTRODUCTION

**Figure 1:**
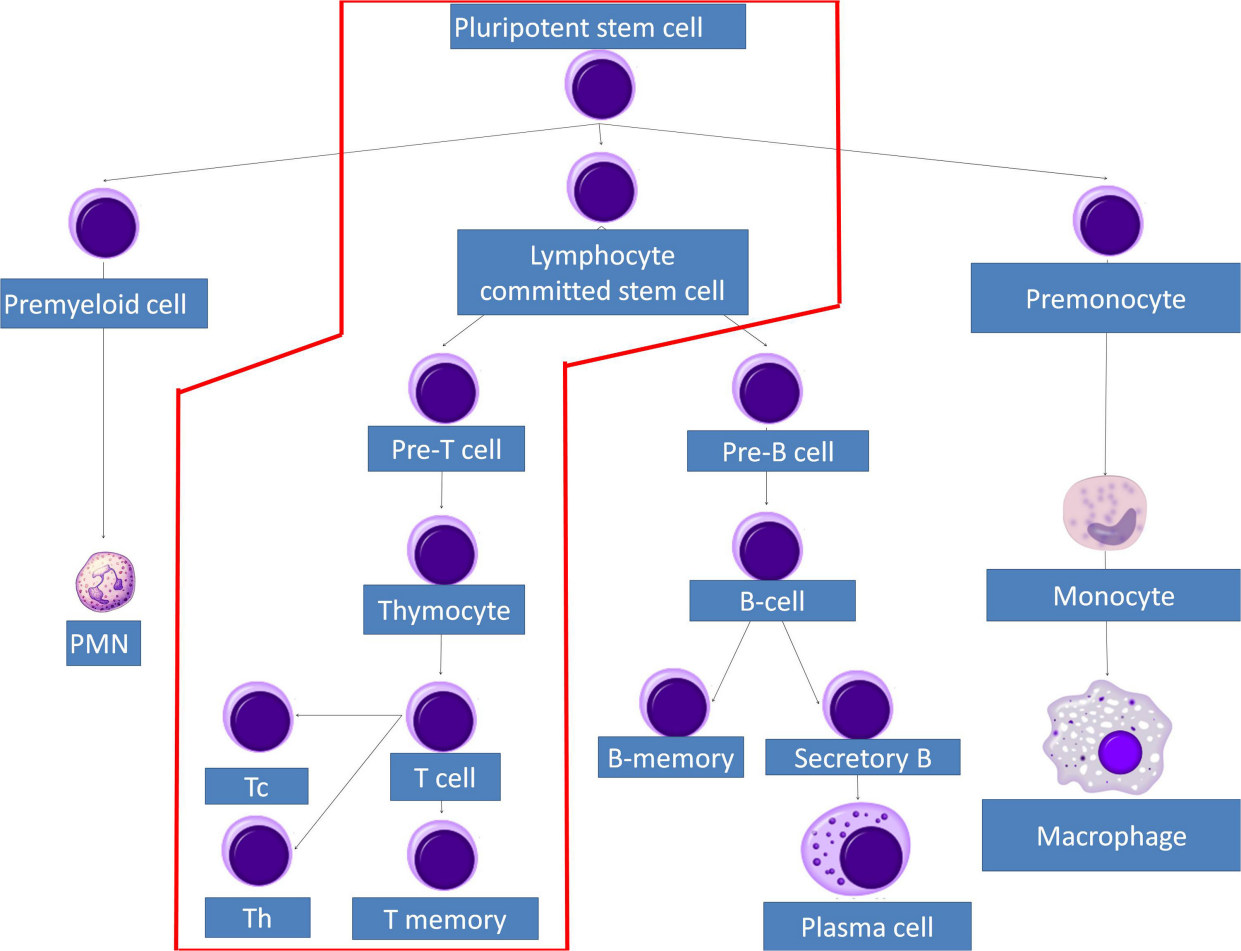
T cell lineage development

**Figure 2:**
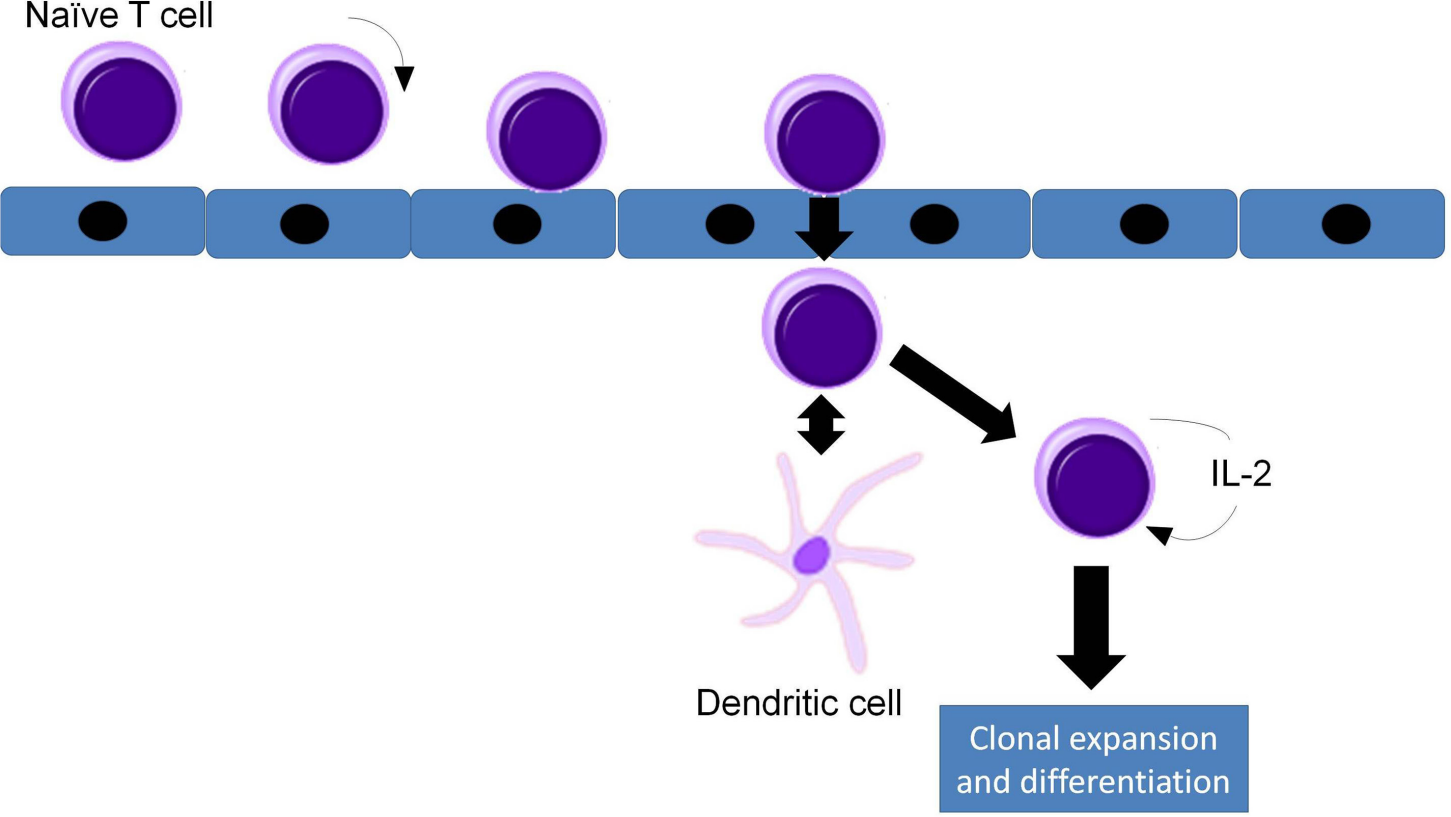
Circulation, rolling and adherence of naïve T cells in the high endothelial venule (HEV), their diapedesis through the HEV wall, interaction with dendritic cells and activation

**Figure 3:**
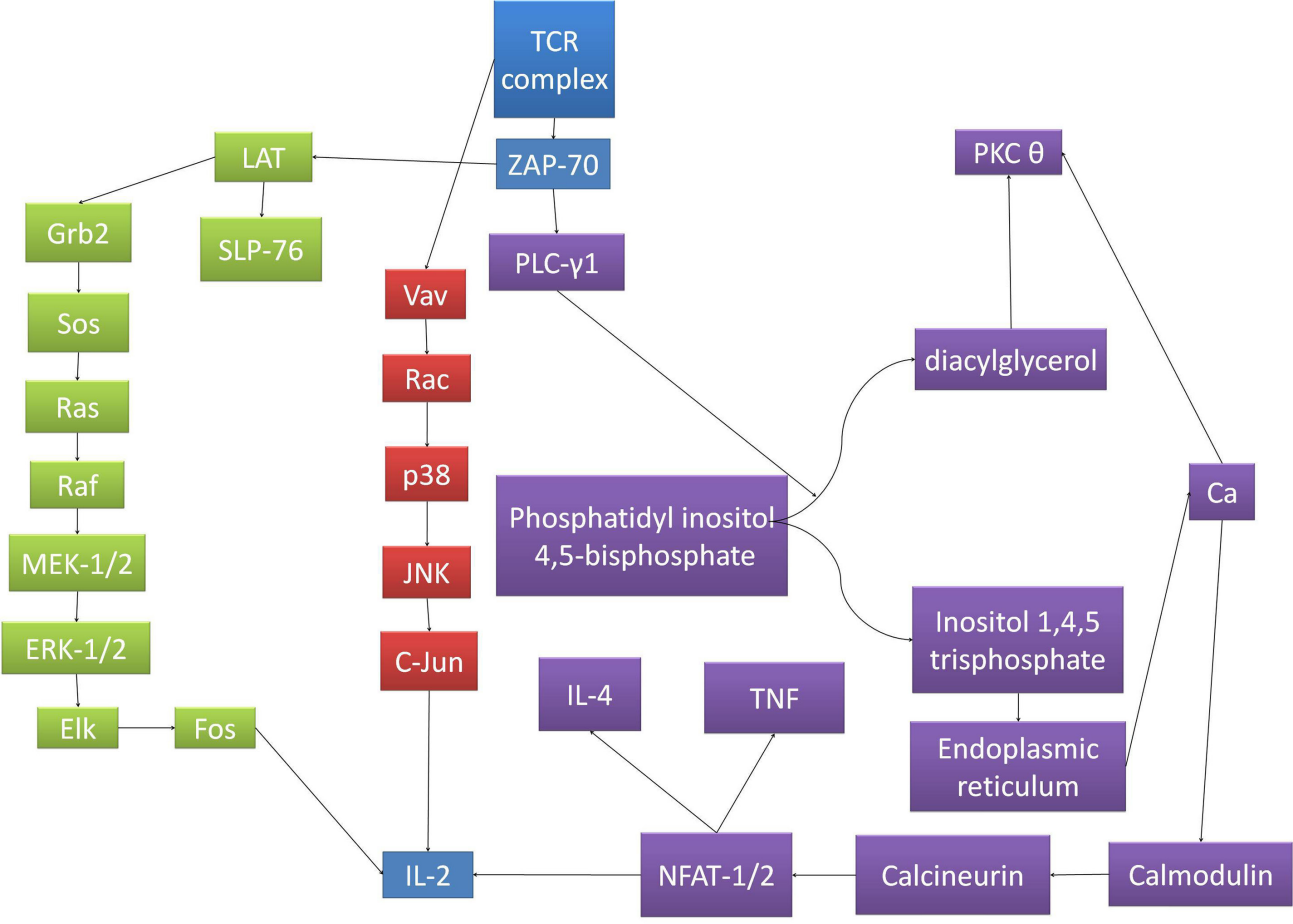
The signaling pathways for TCR complex activation The pathways converge to IL-2 transcription, which determines T cell clonal expansion and subsequent immune response. TCRs functions together with other structures, with which forms the T-cell receptor complex. After the TCR binds the peptide-MHC complex, the TCR undergoes conformational changes that determine the phosphorylation of the ITAMs (immunoreceptor tyrosine-based activation motifs) located on CD247 and the CD3 polypeptides. ITAM phosphorylation creates binding sites for proteins presenting Src homology 2 (SH2) domains, one of the more important ones being the zeta associated protein of 70 kDa (ZAP-70). After binding and activation, ZAP-70 recruits linker of activation of T cells (LAT). After the binding and activation of LAT, other signaling molecules are recruited. Such is the case of SH2-binding leukocyte phosphoprotein of 76-kDa (SLP-76) and Grb2. Grb2 activates SOS, which catalyzes the exchange of GDP to GTP linked to Ras, activating the MAPK pathway, phosphorylating the extracellular receptor-activated kinase 1 and 2 (ERK1/2), which in turn, phosphorylates Elk. Thus determines the transcription of Fos, important for the transcription of interleukin-2 (IL-2). Other pathway that branches off the TCR and phosphorylated proteins complex determine the activation of Vav, which determines the GDP/GTP exchange on Rac, which in turn activates p38. This action determines, in the end, the activation of c-Jun N-terminal kinase (JNK), which phosphorylates c-Jun, representing the second molecule implicated in the transcription of IL-2.

In adults, T lymphocytes start developing from the pluripotent stem cell, go through the stages of lymphocyte committed stem cell, pre-T cell, which migrate in the thymus and form the thymocyte, later migrating to the periphery and forming the naive T cell. This process follows three main paths to T helper cells, T cytotoxic cells and T memory cells (Figures 1 and 2). MHC complexes are recognized by the T cell receptor (TCR), these stimulated T lymphocytes that have an effect on macrophages and B cells, thus augmenting their activities. Thus, a major structures involved in T cell function is the TCR, a receptor with therapeutic potential in the clinic. TCR is formed in more than 90% of the T cells by an α and a β chain. The domains and sequences that form the TCR are (starting from the N-terminus and form the extracellular domains to the intracellular ones): a leader sequence, a variable region, a constant region, a small connecting peptide, a transmembrane domain and a cytoplasmic region [1]. TCRs functions together with other structures, with which forms the T-cell receptor complex, as seen in Figure 3. One topic of high interest is the management of the ICU patient diagnosed with a hematological malignancy, for which he is under treatment. A special emphasis in hematological patients that are treated together with the ICU team is the clinical management based on cellular therapies or immunotherapy. A state-of-the-art protocol that has seen quick development over the last years and presents a high potential in treating hematological malignancies is the CAR-T cell technology [2-4]. Murine models that assess the effects and toxicity of CAR-T cells represent important areas of research before phase I-III clinical trials, due to the potential of this technology to become tomorrow's therapeutics.

CAR-T cells therapy basically requires drawing blood from patients and separating out the T cells. Next, by using a disarmed virus, the T cells are genetically engineered to produce receptors on their surface called chimeric antigen receptors, or CARs. The final step is the infusion of the CAR-T cells into the patient (which is preceded by a “lymphodepletion” chemotherapy regimen). The engineered cells further multiply in the patient’s body and, with guidance from their engineered receptor, recognize and kill cancer cells that harbor the antigen on their surfaces [5-9].

As many other therapeutic alternatives, CAR-T cells also have an important toxicity in the host organism. Nevertheless, the clinical response that CAR-T cells can induce is worth the risk [10]. The toxicity induced by CAR-T cells is linked to immune-mediated adverse effects, out of which some last longer than the toxicities induced by conventional pharmaceutical molecules [10, 11]. Such toxicities are mainly represented by cytokine release syndrome (CRS) and B-cell aplasia [12-16]. The expansion of CAR-T cells and the activation of lymphoid and myeloid cells determine the release of high amounts of pro-inflammatory molecules [17], with the cross-reactivity of CAR-T cells with normal tissues leading sometimes to organ damage, caused by similar expression patterns of normal tissues with the target cells [18, 19]. Furthermore, CAR-T cells and CRS can be also linked by the rise in the levels of IL-6, TNF, IL-2 and IL-8 that eventually lead to arterial hypotension and fever [20-23].

Another important protocol in lymphocyte engineering is the use of DLI for cytomegalovirus (CMV) reactivation, as well as other disorders related to immune deficiency. DLI may also improve the graft-versus-tumor (GVT) or graft-versus-leukemia effect of an allogeneic stem cell transplantation [24, 25]. In the current paper we aim to approach the ICU patient that presents following a diagnosis with a hematological disease and treated mainly through the use of CAR-T cells, DLI, as well as similar novel technologies.

### Design of CAR-T cells

CAR-T cells are produced by a multistep process that implicates primary T cells harvesting, modification and then use. Primary T cells are harvested from a patient’s peripheral blood and enriched. Their enhancement is possible by the use of lentiviral vectors [26-29]. These vectors integrate in the host genome and determine the expression of the CAR construct. One problem that can occur is that the lentiviral capsid presents a natural tropism against CD4+ cells. The solution comes from pseudotyping the lentiviral capsid with viral glycoproteins, the most common used being the vesicular stomatitis virus glycoprotein (VSV-G). However, VSV-G is not suitable for transfection of B and T lymphocytes. Thus, other glycoproteins have been used for this matter, as is the case of the measles virus, hemagglutinin and fusion glycoproteins [30, 31].

Although CAR-T cells have presented great promise for clinical applications, there are two main problems that can arise: CRS and in the case of anti-CD19 CAR-T cells, B-cell aplasia and subsequent immunodepression [23, 32-34]. One of the solutions used in this regard is the use of switch molecules, like rimiducid, to control CAR-T cells activity [35-37]. Another approach for controlling CAR-T cells activity is represented by using suicide genes in the CAR construct [38].

Other concerns regarding CAR-T cells is the potential of lentiviral vectors to generate insertional mutagenesis [39, 40]. Efforts have been made in the scientific community to generate integration-deficient lentiviral vectors and the addition to those of a scaffold/matrix associated region, so the vector and the CAR construct can persist through subsequent cell divisions as an episome [41]. In addition to lentiviral vectors, there are two more approaches that can be used: transposon and the CRISPR/Cas9 system. Transposons have already been used in clinical trials and their insertion in the human genome has not been associated with any disease [42-45]. Nevertheless, if transposons are to be used, a transposase should also be expressed in the cell, either from the same construct or from a different construct, which affects the experimental setup and add more complexity. Inserting the CAR construct in a T lymphocyte by using the CRISPR/Cas9 system is realized from an endonuclease guided by an RNA, each formed from crRNA (the sequence for recognition of the specific part of the genome) and transcrRNA (the sequence that is recognized and bound by the Cas9 so it can be guided at the specific site). This system generates a double stranded break in the host genome and, through, non-homologous end joining the CAR construct can be inserted [46, 47].

Despite CAR-T cell therapy have shown promise in both the preclinical setting, as well as in clinical trials, various drawbacks have been described due to the rapid expansion. One such side-effects is the severe cytokine release after the antigen recognition [32], as well the inability of anti-CD19 CARs to tell the difference between normal and malignant B lymphocytes. This will lead to the development of a long-term B-cell aplasia [23]. Thus, a control of CAR T cell function may be very important due to a reduction of side-effects that might potentially be life-threatening. To overcome this endeavor, various research groups have used antibody-based switch molecules that are designed to control the immunological synapse between a CAR and a malignant cell. This leads to a highly controlled cytotoxic activity, as well as to increased specificity for cancer cells [48, 49].

Another safety concern is related to insertional mutagenesis potential of integrating vectors. Although an important step was made by switching from onco-retroviral vectors to lentiviral vectors, that are considered as a safer alternative to the former ones due to a relative random insertional pattern. However, the oncogenic potential of lentiviral vectors has been previously reported [40, 50] and this might raise safety issues regarding the use of integrating vectors. Towards this end, efforts have been made to reduce the insertional mutagenesis potential of delivery vectors for CAR into T-cells. Generation of integration-deficient lentiviral vectors and inclusion of a scaffold/matrix associated region (S/MAR) in the vector backbone displayed comparable cytotoxic effect of CAR-T cells engineered with non-integrating vectors to those that have the integration function unaffected [51]. Non-integrating vectors due to the presence of S/MAR element in their design are maintained in subsequent cell generation as an episome.

**Figure 4:**
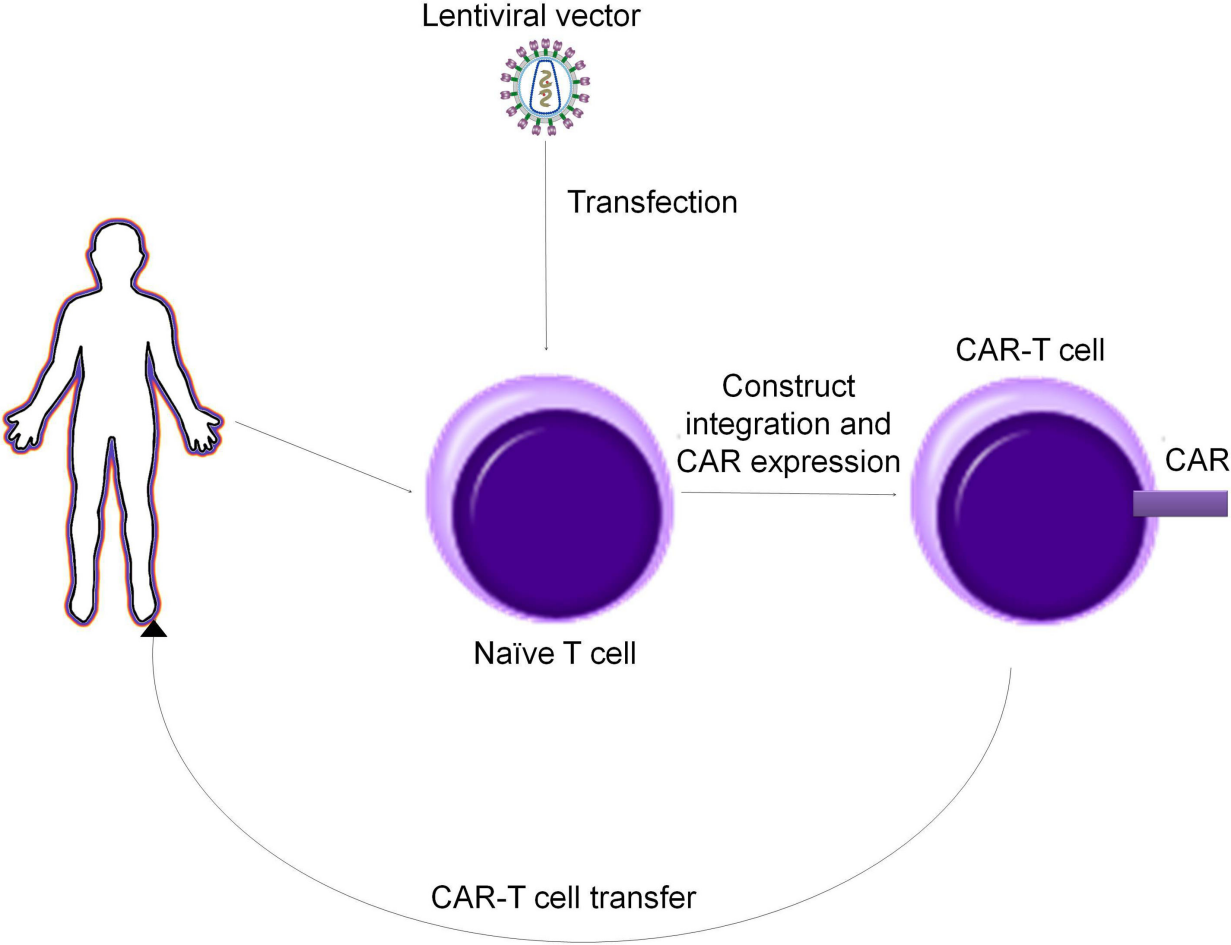
Production alghoritm of CAR T cells

An alternative to lentiviral vectors could be represented by transposons, as they have been described as efficient gene delivery vectors and has been used for gene therapy applications in clinical trials [42, 44], 45]. DNA transposons have been used as gene delivery vehicles instead of retro-transposons because their genomic insertions have not been associated with any human disease [52] (Figure 4). However delivery of the transgene is mediated by an encoding transposase that must be provided in trans from the same construct or a second construct and this might add an extra level of complexity to the experimental setup.

Yet, another alternative to both viral and non-viral delivery could be represented by the newly descried gene editing tool, named CRISPR/Cas9. This technology offers the possibility to target virtually any genomic site in a RNA-guided manner. The editing complex futures the Cas9 nuclease and a guide RNA, comprised of a CRISPR RNA (crRNA) and a trans-acting crRNA (tracrRNA). Upon hybridization of the crRNA to the target sequence, Cas9 generates a double-strand break (DBS), that can be repaired by non-homologous end joining (NHEJ), an event that can result in a loss-of-function of the genomic locus. In the presence of a donor DNA, by a mechanism of homology-directed recombination, an exogenous sequence can be introduced in the targeted locus [46, 47]. This knock-in capability of CRISPR/Cas9 can be exploited to deliver CAR expression cassette in a desired genomic locus that does not interfere with gene function and therefore minimizing the genotoxic effects experienced with integrating viral vectors. And recent improvements in gRNA and Cas9 have reduced the off-target effects to a minimum, increasing the chances of CRISPR/Cas9 to reach clinical applicability. Up to date, CRISPR/Cas9 already proved its applicability in the field of immunotherapy by enhancing CAR-T cells potency by knock-out diverse genes with importance in target recognition and cytotoxic activity [53]. Therefore, CRISPR/Cas9 will surly make a difference in advancing immunotherapies for malignant disorders, in both hematological and solid cancers. However further improvements in delivery systems are still to be made, and stated above, designing more specific and regulated systems are desirable to achieve a controlled activity of CAR-T cells.

In present-day medicine, four generations of CAR-T cells are described, each presenting a chimeric activated receptor with common regions among them. Starting from the exterior of the cell to the cytoplasm these regions are: single-chain variable fragment (scFv), the hinge region, the transmembrane domain and the CD3ζ intracellular domain of the T cell receptor [54, 55]. The first generation of CAR-T cells presented only these regions and showed good results *in vitro*, but did not present efficiency *in vivo* because of the lack of costimulatory signals [56-59]. By trying to overcome these limitations, further generations of CAR-T cells have been developed. The second generation of CAR-T cells present, in addition to the basic construct a costimulatory domain, such as CD28 or 41BB (CD137) close to the CD3ζ domain, which are both associated with clonal expansion and survival of T cells in their activated state [60-62]. The third generation of CAR-T cells can be generated by the addition to the second generation CAR of other costimulatory regions, like CD27, ICOS or OX40 (CD134), which can further improve cell survival [63, 64]. The fourth generation of CAR-T cells (also called TRUCKS) can be built using any of the first three generations and by the addition of a promoter that can be regulated, thus putting CAR-T cell activity under the practitioner’s control [54].

### CAR-T cells-based and DLI therapy in the intensive care unit

Indications of using CAR-T cells therapy are acute lymphoblastic leukemia (ALL), chronic lymphocytic leukemia and non-Hodgkin lymphoma. CAR-T cell therapies are also being developed for solid tumors but studies are being in the early stages. Still, the first steps in investigating the side-effects of CAR T cells are represented by the use of murine models of the therapy. One of the first documented adverse reactions on CAR T cell therapy in preclinical murine models is the cytokine release syndrome (CRS). It has been shown in a murine model that CAR T-cell infusion associated CRS can be prevented through the administration of the kinase inhibitor ibrutinib [16]. To the present date, graft versus host disease (GVHD) is not a real concern regarding CAR T-Cell therapy side effects [65]. In two clinical reports, patients that underwent allogeneic hematopoietic stem cell transplant (allo HSCT) also received infusions of anti CD19 CAR allogeneic T cells from their initial transplant donors. The first report did not identify any GVHD in any of the eight transplanted patients [66], while the second report showed that one out of twenty patients developed a worsening of a pre-existing chronic GVHD [67].

Across the large variety and number of preclinical publications focusing on CAR T cells, very few of them document toxicity in animal models as it would seem normal with any new compound that has a potential use in a clinical setting. Paradoxically, there are numerous studies reporting the clinical use of CAR T cells even though their safety has not yet been evaluated extensively *in vivo*. An explanation for this phenomena could include factors like the large variety of engineered CAR cells, the differences between mouse and human physiology and T-Cell biology and the differences in drug metabolism capacity in each species. An example which would confirm this hypotheses would be the fact that one *in vivo* study involves CAR T cells targeting the Her2/neu antigen, proving the antineoplastic activity and the biological safety of Her2/neu-specific CAR T cells in transgenic animals with lymphodepletion [68], yet the clinical trial involving the same engineered cells showed that one of the patients died due to a massive cytokine release syndrome [69]. The majority of preclinical studies investigating CAR T cells have focused on verifying their specificity and potency for antineoplastic activity, the key advantage of CARs *in vivo* being the fact that they possess the ability to redirect T-cell effector function without HLA-restriction. The *in vivo* testing of CARs expresses several drawbacks. First of all the successful engraftment of T-cells in immunocompromised mice is hard to achieve due to the residual elements of the mouse’s innate immune system; another drawback is the fact that even if the engraftment is successful, most of the mice develop GVHD in long term studies (more than 60 days) [70]. CAR T-Cells target human antigens which are restricted to transplanted tumor cells in mice, rendering the assessment of their effects on healthy tissues in mice models hard to achieve [71]. The humanized NSG mouse has been an indispensable tool for evaluating short-term CAR T cell activity *in vivo*. CARs that act against ROR1 for mantle cell lymphoma and CD44v6 for acute myeloid leukemia and multiple myeloma have been tested in humanized NSG mice extensively [72, 73]. Humanized mice have been also used to assess the function and efficacy of co-stimulatory domains like CD27, ICOS, CD28 and 4-1BB, due to their potential enhanced efficiency in targeting malignancies and augmenting CARs safety [74, 75]. In a humanized animal model, the Hu-PBL-SCID NSG mouse, engineered T cells showed the ability to destroy a cancer cell line that expressed prostate tumor antigens [76]. Modern genetic engineering methods like messenger RNA transduction have been used to generate CAR NK-Cells and to successful target a non-Hodgkin's lymphoma in a Hu-PBL-SCID NSG model. The study confirmed that activated expanded PBNK became highly cytolytic, eradicating resistant CD20+ B-leukemia/lymphoma after nucleofection with anti-CD20 CAR messenger RNA [77]. Najima *et al* have successfully transplanted WT-1 specific TCR transduced human HSCs into class I matched transgenic NSG mice. The WT-1 tetramer positive T cells differentiated in the thymus and the splenic cytotoxic lymphocytes of the mice targeted leukemia cells in an antigen-specific HLA restricted manner and destroyed them [78].

Even if current mouse models for CAR T cells have a poor predictive nature, these may relate to the biological differences between species, a barrier which could be overcome by developing new humanized mice models. Studies in the last decade have focused mainly on their clinical applications with toxicity being neglected as a main research aim. Most of the clinical studies report toxic effects on these engineered cells which in turn will cause a stronger will of the researchers to better understand the potential mechanisms of *in vivo* toxicity by developing better animal models to this purpose.

Cytokine capture system (CCS) is a protocol for isolating different cell populations, stimulating and using them further on. However, the manual method of CCS is time consuming, requiring 10 to 12 hours and has to be undertaken by a skilled operator. These problems have been solved by Prodigy (Miltenyi Biotech), an apparatus that can perform this operation in a closed environment and within 2 hours. The automated protocol consists in four main steps: preparation of materials under sterile conditions, preparation and use of automated cell enrichment system, cell count determination and examination of the separation performance; these steps are described in more detail by Kumaresan *et al* [79]. The use of this technique, and thus of Prodigy, has been studied in the field of hematopoietic stem cell transplantation (HSCT), specifically offering an infusion of selected T cells which have an effect against opportunistic infections and also improve the GVT effect [80, 81]. A good example of using this assay is the treatment of opportunistic CMV infections in patients that have underwent an allogeneic HSCT. This can be done by incubating overlapping peptides from the CMV pp65 antigen with total nuclear cells provided by CMV positive donors. The pp65 peptide fragments stimulate the T cells to secrete interferon-γ (IFN-γ), after which, antigen-specific T-cells can be selected, using reagents that can recognize CD45 and IFN-γ [82-84].

DLI represents a form of adoptive therapy used after allogeneic stem cell transplant for its anti-tumor and anti-infectious properties and it has been used to restore the patient’s immune function, thus aiding in the prophylaxis or treatment of relapse, in preventing infectious diseases (as is the case of CMV infection) and to establish full donor chimerism [85-87]. The start of the idea of GVT activity was observed after a flare of graft versus host disease (GVHD) or after sudden discontinuation of immunosuppression [88, 89]. GVT can be confirmed by the fact that T cell-depleted graft transplantation are associated with a higher risk of relapse [90-92]. Following these observations, DLI was tested for its antileukemic effects. The first described application was in 1990 by Horowitz *et al*, who described three patients treated for chronic myelogenous leukemia (CML) with matched-sibling allogeneic HSCT that have relapsed with chronic-phase CML and were treated for the relapse with DLI. The results showed a complete cytogenetic remission in all three patients. Further studies were done for the use of DLI against CML relapse and the majority of the studies have presented long term molecular remission. DLI has been studied as a therapeutic tool in other hematological malignancies, but it’s efficiency was not as good as with CML [93-97].

The complications that occur after DLI include, more notably GVHD and marrow aplasia. GVHD in these patients has been correlated with a GVT effect, although some studies contradict these results [93, 95, 98-101]. Although progress has been made in predicting GVHD, the influence of DLI on these effects remains poorly defined. In this direction, studies working on the hypothesis that lower doses or escalating doses of T cells can minimize GVHD, while still maintaining a significant level of GVT activity, have been made [102-104]. Even if the advantage of unrelated donor grafts depleted of T cells is obvious through the lower incidence of GVHD, there are also marked disadvantages to this approach, one of them being represented by the increased number of opportunistic infections CMV and Epstein-Barr virus infections, but not only [105-111]. Naturally, several studies have been devised to research the application of DLI in preventing or curing these infections [110-114]. A study on 462 recipients of bone marrow transplant from unrelated donors showed that infections were the cause of 30% of deaths, compared to 14% in the case of disease recurrence [115]. Other reports showed that infections accounted for 38 to 75% of the cases of death compared to 8 to 25% in the case of leukemia relapse [108, 109, 116-118]. One study, in particular, has analyzed the immune reconstitution after unrelated HSCT, in this report is has been show that T cell depleted HSCT, is associated with prolonged T cell lymphopenia and CD4 lymphopenia [119-122].

Other refinements or reconfigurations of CAR-T cells are being tested. One approach is the development of CAR-T cells therapies that use immune cells collected not from patients, but from healthy donors. The idea is to create so-called off-the-shelf CAR-T cells therapies that are immediately available for use and don’t have to be manufactured for each patient.

Although adoptive transfer of CAR-T cells is a unique and promising cancer therapeutic, there are significant safety concerns. Clinical trials of this therapy have revealed potential toxic effects of these CARs when healthy tissues express the same target antigens as the tumor cells, leading to outcomes similar to GVHD [65, 123-125]. A potential solution to this problem is engineering a suicide gene into the modified T cells. In this way, administration of a prodrug designed to activate the suicide gene during GVHD triggers apoptosis in the suicide gene-activated CAR-T cells. This method has been used safely and effectively in HSCT. Adoption of suicide gene therapy to the clinical application of CAR-T cells adoptive cell transfer has potential to alleviate GVHD while improving overall anti-tumor efficacy [66, 67, 126].

Early case reports of unexpected organ damage and deaths following CAR-T cells therapy first highlighted the possible dangers of this new treatment. CAR-T cells can potentially damage normal tissues by specifically targeting a tumor-associated antigen that is also expressed on those tissues.

### Complications that require ICU admission for the CAR-T cells-treated patient

The main complications for patients that receive a CAR-T cells-based therapy are the CRS and B-cell aplasia, out of which CRS is one of the most severe, requiring ICU admission and treatment. It ranges from mild to life threatening and it is an oncologic emergency. CRS can be observed following the previous administration of immune-based therapy drugs, as is the case of rituximab or other monoclonal antibodies [127, 128]. This condition appears due to a massive release of cytokines (high levels of IL-6 and IL-12) into the bloodstream, followed clinically by high fever and a sudden fall in blood pressure, tachycardia, as well as hemophagocytic lymphohistiocytosis or macrophage-activation syndrome [129], even multi-organ failure with potential fatal outcome [130, 131]. IL-6 is important in neutrophil trafficking, acute phase response, B-cell differentiation, angiogenesis and the production of autoantibodies and is produced by dendritic cells, monocytes, T cells, keratinocytes and fibroblasts. IL-10 also plays an important role, being produced by monocytes/macrophages and it regulates both cell-mediated and innate immunity after the inhibition of the activated macrophages. This interleukin is synthethised by B cells, mastocytes and T helper cells, but not by cytotoxic T cells, thus making it not the ideal target cytokine for treatment options in CRS. Other molecules involved in CRS are the TNF, IL-8 and IL-2, reported in patients treated with CAR-T cells and blinatumomab [132-135].

In a patient with newly developed fever, which is often the first sign, CRS is diagnosed as the day with the first fever over 38 °C related to the infusion of CAR-T cells and the recovery from CRS is diagnosed as 24 hours without fever or vasoactive medication [131, 136, 137]. The clinical management of CRS is oriented according to the grade, as presented in Table 1.

**Table 1:** Clinical grading of cytokine release syndrome

Grade	Clinical symptoms / Therapy
Grade 1	Not life-threatening symptoms. Only symptomatic therapy is advised – intravenous fluids, antipyretics
Grade 2	Symptoms that require moderate intervention. Oxygen requirement <40% or arterial hypotension treated with fluids or low dose vasopressor or grade 2 organ toxicity
Grade 3	Symptoms that require aggressive intervention. Oxygen requirement >40% or arterial hypotension treated high dose vasopressors or grade 3 organ toxicity or grade 4 transaminitis
Grade 4	Life-threatening symptoms, that require ventilator support or grade 4 organ toxicity, with the exception of transaminitis
Grade 5	Death of the patient

Cytokine elevations are measurable in most patients and a CRS approach may be by targeting elevated cytokine directly with anti-cytokine directed therapies, as is the case of tocilizumab. This drug is a humanized monoclonal antibody directed against the IL-6 receptor and its use has lead to a dramatic improvement of severe CRS for patients treated with CAR-T cells or blinatumomab (a drug used for Philadelphia chromosome-negative relapsed or refractory acute lymphoblastic leukemia). Still, tocilizumab comes with side-effects such as elevated liver enzymes and cytopenias. IL-6 is a potential target for other new inhibitors, as is the case of siltuximab and the IL-6 trans-signalling blocker sgp130Fc. These small molecules are interesting at this point, but further phase I-III clinical trials are required [138-140].

The use of corticosteroids represents an obvious solution for CRS because they have proven their efficacy in inhibiting activated T cells, as is the case of GVHD. The major drawback that limits their use could be the potential to negatively affect the antitumor effects of the CAR-T cells. Corticosteroids have shown a partial response in a patient who received corticosteroids early after the infusion of CAR-T cells [132, 141, 142]. Brentjens et al have used short-term steroids for the therapy of severe CRS without compromising CAR-T cell proliferation and efficiency. At least *in vitro*, the steroids take down the cytokine levels without the reduction of T-cell activation, but *in vivo* the effects may be totally different. Thus, the use of corticosteroids should be reserved for neurological symptoms and CRS unresponsive to tocilizumab [71, 143-145].

Steroids still represents the basic therapy for blinatumomab-induced CRS, as the Food and Drug Administration (FDA) recommends pretreatment with 20 mg of intravenous dexamethasone before the first dose of blinatumomab, as well as before each intracycle dose escalation, as well as when restarting an infusion after the previous interruption of therapy.

CRS management should involve the entire healthcare professional team, not only the hematologist or the ICU doctor, but also the nursing team. Nurses must thus be familiar with the toxicity profile, the type of infusion and with the protocols used in monitoring the vital signs for any indication of a severe reaction [146-148].

Taking all this into consideration, patients receiving CAR-T cell therapies should have limited comorbidities so that they are able to tolerate potentially severe CRS.

## CONCLUSIONS

T lymphocytes play an important role in the treatment of cancer. T cells behavior are influenced by the T-cell receptors complex and calcium signaling. CAR-T cells and DLI are novel diagnostic and treatment technologies for hematological malignancies in intensive care units. CAR-T cells are classified as first, second, third and fourth generation cells, depending on the intracellular signaling domain number of T cell receptors. Clinical trials utilizing the first generation CAR-T cells have failed to exhibit significant clinical benefits because lack of costimulatory signals so further generations of CAR-T cells have been developed by trying to overcome this limitation. Although CAR-T cells have presented great promise for clinical applications, there are significant safety concerns. The main complications are the cytokine release syndrome and B-cells aplasia. Another important protocol in lymphocyte engineering is the use of DLI which represents a form of adoptive therapy used after stem cell transplant for its anti-tumor and anti-infectious, thus aiding in the prophylaxis or treatment of relapse, in preventing infectious diseases and to establish full donor chimerism. The complications that occur after DLI include GVHD and marrow aplasia. Preventive strategies which could include using predictive biomarkers for predicting which patients will become critically ill should be researched.

Further studies are necessary to establish clear guidelines for treating hematological malignancies with these therapies and a better collaboration between hematologists and intensive care unit doctors.

## Abbreviations

CAR-T cells: chimeric antigen receptor-T cells
DLI: donor lymphocyte infusion
ICU: intensive care unit
CRS: cytokine release syndrome
GVT: graft-versus-tumor
GVHD: graft versus host disease
