# Correction: Genetically enhanced T lymphocytes and the intensive care unit

**DOI:** 10.18632/oncotarget.25983

**Published:** 2018-08-10

**Authors:** Tiberiu Tat, Huming Li, Catalin-Sorin Constantinescu, Anca Onaciu, Sergiu Chira, Ciprian Osan, Sergiu Pasca, Bobe Petrushev, Vlad Moisoiu, Wilhelm-Thomas Micu, Cristian Berce, Sebastian Tranca, Delia Dima, Ioana Berindan-Neagoe, Jianliang Shen, Ciprian Tomuleasa, Liren Qian

**Affiliations:** ^1^ Intensive Care Unit, Ion Chiricuta Clinical Cancer Research, Cluj Napoca, Romania; ^2^ Department of Anesthesiology-Intensive Care, Iuliu Hatieganu University of Medicine and Pharmacy, Cluj Napoca, Romania; ^3^ Department of Pulmonary and Critical Care Medicine, Navy General Hospital of PLA, Beijing, China; ^4^ Department of Hematology, Iuliu Hatieganu University of Medicine and Pharmacy, Cluj Napoca, Romania; ^5^ Research Center for Functional Genomics and Translational Medicine, Iuliu Hatieganu University of Medicine and Pharmacy, Cluj Napoca, Romania; ^6^ Department of Experimental Medicine, Iuliu Hatieganu University of Medicine and Pharmacy, Cluj Napoca, Romania; ^7^ Department of Hematology, Ion Chiricuta Clinical Cancer Research, Cluj Napoca, Romania; ^8^ Department of Hematology, Navy General Hospital of PLA, Beijing, China; ^9^ Research Center for Functional Genomics and Translational Medicine / Hematology, Iuliu Hatieganu University of Medicine and Pharmacy, Cluj Napoca, Romania

**This article has been corrected:** The correct Acknowledgment information is given below:

**ACKNOWLEDGMENTS**

Tiberiu Tat, Huming Li and Catalin Constantinescu contributed equally to the current paper. The research on CAR-T cells was funded by an internal grant of the Iuliu Hatieganu University, awarded to Tiberiu Tat, as well as by a National Grant of the Romanian Government, for postdoctoral research, awarded to Delia Dima. This paper was financed by a grant awarded to Ciprian Tomuleasa - FDI - 2018.

Original article: Oncotarget. 2018; 9:16557-16572. https://doi.org/10.18632/oncotarget.24637

